# A cross-sectional study of the prevalence of intensity of infection with *Schistosoma japonicum *in 50 irrigated and rain-fed villages in Samar Province, the Philippines

**DOI:** 10.1186/1471-2458-6-61

**Published:** 2006-03-09

**Authors:** Mushfiqur R Tarafder, Ernesto Balolong, Hélène Carabin, Patrick Bélisle, Veronica Tallo, Lawrence Joseph, Portia Alday, Ryan O'Neil Gonzales, Steven Riley, Remigio Olveda, Stephen T McGarvey

**Affiliations:** 1 Department of Biostatistics and Epidemiology, College of Public Health, University of Oklahoma Health Sciences Center, Oklahoma, USA; 2 Research Institute for Tropical Medicine, Alabang, Muntinlupa City, Philippines; 3 Division of Clinical Epidemiology, Montreal General Hospital, McGill University Health Center, Montreal, Quebec, Canada; 4 Department of Epidemiology and Biostatistics, Faculty of Medicine, McGill University, Montréal, Québec, Canada; 5 International Health Institute, Brown University, Providence, Rhode Island, USA; 6 Department of Community Medicine, Faculty of Medicine, The University of Hong Kong, Hong Kong

## Abstract

**Background:**

Few studies have described heterogeneity in *Schistosoma japonicum *infection intensity, and none were done in Philippines. The purpose of this report is to describe the village-to-village variation in the prevalence of two levels of infection intensity across 50 villages of Samar Province, the Philippines.

**Methods:**

This cross-sectional study was conducted in 25 rain-fed and 25 irrigated villages endemic for *S. japonicum *between August 2003 and November 2004. Villages were selected based on irrigation and farming criteria. A maximum of 35 eligible households were selected per village. Each participant was asked to provide stool samples on three consecutive days. All those who provided at least one stool sample were included in the analysis. A Bayesian three category outcome hierarchical cumulative logit regression model with adjustment for age, sex, occupation and measurement error of the Kato-Katz technique was used for analysis.

**Results:**

A total of 1427 households and 6917 individuals agreed to participate in the study. A total of 5624 (81.3%) participants provided at least one stool sample. The prevalences of those lightly and at least moderately infected varied from 0% (95% Bayesian credible interval (BCI): 0%–3.1%) to 45.2% (95% BCI: 36.5%–53.9%) and 0% to 23.0% (95% BCI: 16.4%–31.2%) from village-to-village, respectively. Using the 0–7 year old group as a reference category, the highest odds ratio (OR) among males and females were that of being aged 17–40-year old (OR = 8.76; 95% BCI: 6.03–12.47) and 11–16-year old (OR = 8.59; 95% BCI: 4.74–14.28), respectively. People who did not work on a rice farm had a lower prevalence of infection than those working full time on a rice farm. The OR for irrigated villages compared to rain-fed villages was 1.41 (95% BCI: 0.50–3.21).

**Discussion:**

We found very important village-to-village variation in prevalence of infection intensity. This variation is probably due to village-level variables other than that explained by a crude classification of villages into the irrigated and non-irrigated categories. We are planning to capture this spatial heterogeneity by updating our initial transmission dynamics model with the data reported here combined with 1-year post-treatment follow-up of study participants.

## Background

Schistosomiasis japonica is a parasitic zoonosis and an important public health problem in the Philippines. The disease was first reported in the Philippines in 1906 [1]. It has been estimated that 6.7 million people live in areas endemic for *Schistosoma japonicum *which includes 24 provinces, 183 municipalities, and 1212 villages [1]. The three major focal points of disease transmission are located on the regions of the Visayas and Mindanao [2]. The life cycle of *S. japonicum *includes an amphibious snail of *Oncomelania *species and a mammalian definitive host, including humans or other animal reservoirs. Unlike other species of schistosomes, animal reservoirs are thought to contribute significantly to the transmission and maintenance of life cycle of *S. japonicum *in areas where it is endemic [2]. Rice farming and annual rainfall patterns in these endemic areas maximize contact among humans, animal reservoirs and freshwater infested with *Oncomelania *snails which in turn increase the risk of infection [2,3]. In addition, according to the severity of histopathology and intensity of infection, *S. japonicum *is thought to be more pathogenic than the other schistosomes [4-6].

In the Philippines, national control programs conduct surveys periodically to estimate the prevalence of infection, the level of endemicity and the need for sustained control programs in each region. These surveys are based on the Kato-Katz technique of only one stool sample per person, which has been shown to heavily underestimate the true prevalence of infection [6-9]. Also, it has been shown that control programs can have a substantial impact on intensity of infection even when no significant change is observed in prevalence of infection [10].

In the Philippines, relatively few studies have reported the intensity of human infection, as expressed by the number of eggs per gram (epg) of stool [4,11-14]. When this is done, assumptions of normality may not be met and even the reporting of geometric means may not be appropriate. Another issue with parasitic infections is the fact that prevalence and intensity of infection is often clustered at the village level [15]. Although there are several papers on day-to-day and intra-specimen variations in the intensity of *S. japonicum *infection, only Hubbard et al. (2002) and Spear et al. (2004) provide data on village-to-village variation in the intensity of infection in a *S. japonicum *endemic region of China [16,17]. Such variation has not been reported in the Philippines. This study describes the prevalence, intensity and village-to-village variation in intensity of infection across 50 villages of Samar Province (also known as Western Samar), the Philippines. These results are based on data measured as a baseline for a longitudinal study which will be used to develop dynamic mathematical models of transmission and control [18].

## Methods

The description of the village-to-village variation in the prevalence of two levels of intensity of infection with *S. japonicum *was part of a larger project which aimed at assessing the effect of water management systems on the transmission dynamics of the infection. The project was designed to answer this particular question and this explains the sampling approach adopted (see below).

### Study area

This cross-sectional study was conducted between August 2003 and November 2004 in the Province of Samar, the Philippines. According to the data from the national schistosomiasis control program, 13 municipalities and 134 villages (known locally as barangays) were endemic for *S. japonicum *in Samar Province (Martinez, personal communication, 2003). In 2002, there were approximately 101,954 hectares of farmland in Samar Province [19].

### Selection of villages

The following eligibility criteria were applied to the 134 endemic villages 1) Safety of field team; 2) relatively accessible; 3) at least 50 households in the village; and 4) not located near seacoast nor a peri-urban village. This left 75 eligible for participation in the study. All these villages are rural rice farming communities. These 75 villages were classified as either rain-fed or irrigated, based on assessments of their irrigation and farming characteristics, based on interviews and on ocular inspection of the villages. Farmers and/or village leaders were asked about the number of hectares of rice farming irrigated by man-made water management systems and the number of hectares rain fed.

The following criteria were used to select the 25 rain-fed villages: 1) 90 – 100% of rice farming hectares in the village are rain fed; 2) the absence of National Irrigation Administration projects or Department of Agriculture assisted pumps or dams; 3) the absence of polyvinyl chloride or cemented canals; and 4) a minimum of 15 hectares of rice farming. The 25 irrigated villages were chosen as follows. Fifteen (15) villages were chosen based on 1) the presence of sophisticated irrigation systems and a minimum of at least 7 irrigated hectares and 2) at least 20% of the total hectares were irrigated. Ten (10) additional villages were chosen based having the largest area of irrigated farm land.

### Selection of households within villages

In each village, a maximum of 35 eligible households were randomly selected. This number was a compromise between feasibility and obtaining a sufficient number of households to fit hierarchical models. In all villages, eligible households had to include at least five people. In rain-fed villages, households where at least one household member worked full-time in a rain-fed farm were eligible for selection. In irrigated villages, households where at least one household member worked most of the time in an irrigated farm were eligible for sampling. When 35 or fewer households were eligible in a village, they were all invited to participate in the study. The head of each household was asked for his/her consent to participate in the study. Information on the reason for a household head to refuse participation was recorded. When this occurred, the head of the next available and eligible household was invited to participate. The head of each household was also asked questions related to the household environment, its members and the presence and use of domesticated animals.

### Selection of participants with households

At most six individuals including at least one farmer were selected at random from each household for a maximum of six participants per household. All selected household members were asked for their consent to participate in the study. Only those individuals who consented were included. Parents of children aged less than 18 were asked for their consents and children aged 7 and more were asked for their assent. Information on individuals refusing to participate was collected. When an individual refused to participate, s/he was replaced by another consenting household member. An individual-level interview was conducted and included questions on socio-demographic aspects such as age, sex, occupation and on health history regarding schistosomiasis. The study was approved at its inception by the Institutional Reviews boards of Brown University and of the Research Institute for Tropical Medicine and by the ethics committee of the Danish Bilharziasis Laboratory.

### Measurement of infection with S. japonicum

Each participant was asked to provide three stool samples over consecutive days. Though the goal was to have at least two stool samples per participant, all individuals who provided at least one day stool sample were included in the analysis. All stool samples were processed in the field. Each slide was examined for *S. japonicum *eggs using 2-slides Kato-Katz thick smear technique [20]. The numbers of eggs on each slide were summed, the total divided by two and multiplied by 20 to obtain the number of epg per stool sample.

### Statistical analysis

A Bayesian three outcome category cumulative logistic regression model with a hierarchical component was used for analysis. The outcome data are epg counts from the stool samples collected over a maximum of three days for each participant clustered within villages. The epg counts are categorized into uninfected (0 epg), light infection (1 to 100 epg) and moderate to heavy infection (over 100 epg). These categories were chosen because no participants were found to be even moderately infected in 14 villages, only a small number of individuals (68) had heavy infection (more than 400 epg) and even fewer individuals (30) had 800 epg or more. This distribution of the epg data led us to combine the categories of those with heavy and moderate infections. Based on the epg results within each participant over three days, the model was used to estimate the probability that each participant's infection status falls within a given category of epg counts. The first level of the hierarchical logit model estimates most likely epg count category for each participant and includes one intercept parameter for each village, as well as independent variables for occupation, age, sex and interaction between age and sex. Occupation is used as a categorical variable and includes working most of the time on a rice farm (reference category), working some of the time on a rice farm, working on a farm but never on a rice farm; not working on a farm; not working on a rice farm but may be working on another type of farm; being aged 10 years old or less. The category "working on a farm but never on a rice farm" includes individuals who had declared non-rice farming as their usual occupation and had declared not having worked in a rice farm in the past 9 months. The category "not working on a rice farm but may be working on another type of farm" were individuals going to school (more than 10 years old) who said that they performed chores for their parents or who did part time jobs when not at school and who also declared not having worked on a rice farm in the past 9 months. It also included non students who did not mention their usual occupation and declared that they had not worked on a rice farm in the past 9 months. This later group of people are hence individuals who did not work on rice farms but who could have worked on non rice farms. Age is divided in four categories: 0–10 years old (reference), >10 to 16 years old; >16 to 40 years old and more than 40 years old. At the second level of our hierarchical model, the intercept parameters from each of the 50 villages were modeled as a linear regression. These intercepts allow for between village heterogeneity in the prevalence proportions of light and at least moderate infections, and represent the overall prevalence levels in each village, adjusted for individual level covariates. At the third level, vague prior distributions were specified for all of our unknown parameters. For example, all regression coefficient parameters were given normal distribution priors, centered at zero and with variance of 10,000, and all standard deviation parameters were given uniform distributions over a wide range. We report the predicted proportion and 95% Bayesian credible interval (BCI) of individuals lightly and at least moderately infected in each village. Methods used in this paper to adjust for the imperfect measurement of degree of infection were similar to those used in a previous paper [23] by this research group (Schistosomiasis Transmission Ecology Project in the Philippines (STEP)). The program written in WinBugs is available from the authors on request.

## Results

A total of 1427 households, of which 132 (9.3%) were replacements, were included in the study. Reasons given for non-inclusion of households were ineligible households (51.5%), refusal to participate (15.2%), the household members were not permanent residents of the village (12.9%), the household members migrated elsewhere (11.4%), no one could be reached after at least 3 attempts (6.1%) and the household head lived outside the home (3.0%). A total of 175 (2.6%) household members had to be replaced because they were not willing or did not want their children to participate (21.1%), they were no longer living in the selected household (65.1%), they were physically and mentally incapacitated (6.9%) or for other reasons (6.9%).

Out of 6917 individuals who agreed to participate in the study, 5624 (81.3%) provided at least one stool sample for analysis. The proportions of male participants who provided at least one stool sample was 52.8% and 56.1% among those who did not provide any stool sample. The mean ages of participants who provided at least one stool sample and those who didn't provide any stool sample were 24 years (standard deviation (SD): 19.06) and 22 years (SD: 16.16), respectively. Minimum and maximum ages were the same for both groups, 2 months and 85 years, respectively. Among those who provided at least one stool sample, 40.2% worked most of the time on a rice farm, 36.6% were 10 year old or younger children, 14.4% did not work on a rice farm but may have worked on another type of farm, 3.8% worked sometimes on a rice farm, 3.7% did not work on any farm and 1.4% worked on a farm but not on a rice farm. Among those who did not provide any stool sample, the percentages of different occupations were 39.3% (most of time on rice farm), 5.3% (sometimes on rice farm), 1.6% (on non-rice farm), 6.7% (no farming), 21.5% (not rice farm, maybe other farm) and, 25.7% (10 year old or less), respectively.

**Figure 1:**
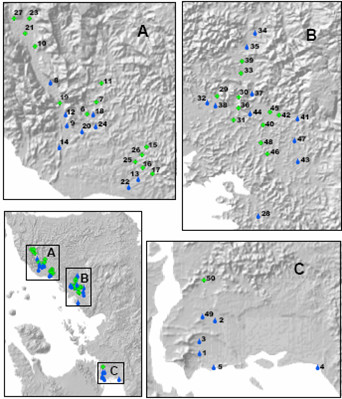
Location of the 50 study villages and their irrigation status in three geographical areas (A, B and C) of the Province of Samar, the Philippines. Green cross: irrigated villages. Blue drop: rain-fed villages. Numbers beside each point correspond to the village numbers in Table 1.

Figure 1 illustrates the location of each study village and their irrigation status. Table 1 shows socio-demographic data on the study participants who provided at least one stool sample according on the type of village they lived in. Table 1 clearly shows that there were no major differences in the age distribution, sex, occupation and number of stool samples collected between people living in rain-fed villages and those living in irrigated villages. It also confirms the fact that our study villages are largely rice farming communities. Among adults and children not attending school, 70% of the villagers in the irrigated and rain-fed villagers identified rice farming as their primary occupation, with only 15% declaring themselves of not being farmers (mostly females staying at home).

Table 2 shows the proportion of individuals who provided at least one stool sample and the proportion of individuals with different intensities of infection for each village. The predicted proportion of individuals lightly and at least moderately infected were 17.7% (95% BCI: 15.3%–20.2%) and 3.1% (95% BCI: 2.2%–4.7%), respectively. The proportion of participants who were lightly infected varied from 0.0% (95% BCI: 0.0%–3.1%) to 45.2% (95% BCI: 36.5%–53.9%) from village to village. For those who were at least moderately infected, the proportion varied from 0.0% (95% BCI: 0.0%–1.6%) to 23.0% (95% BCI: 16.4%–31.2%). These overall results must be interpreted with care as the intensity of infection varied remarkably from village to village (Table 2).

Table 3 shows the probability that the individuals were classified in the non-infected, lightly infected or at least moderately infected categories given their predicted category of infection. The average probability of being classified as not infected when most likely not infected was excellent at 99.3% (95% BCI: 98.8%–99.8%). However, the average probabilities of being classified as not infected when most likely lightly or at least moderately infected were 57.9% (95% BCI: 52.2%–64.1%) and 12.3% (95% BCI: 5.1%–20.7%), respectively. In addition, the average probability of being classified as lightly infected was 26.5% (95% BCI: 17.5%–35.9%) when most likely at least moderately infected (Table 3).

Table 4 shows odds ratios (ORs) estimated using the cumulative-logit model with the above adjustments for measurement error. The ORs compare individuals at least lightly infected (>0 epg) to individuals not infected (0 epg) and individuals moderately or heavily infected (>100 epg) to individuals lightly infected or not infected (0–100 epg). Sex modified the effect of age on the probability of being in the two infected categories. Using the 0–7 year old group as a reference category, the highest OR among males was that of being aged 17–40-year old, 8.76 (95% BCI: 6.03–12.47). Among females, the age group with highest OR was 11–16-year age group, 8.59 (95% BCI: 4.74–14.28). Thus, females tended to get higher intensities of infection at a younger age than males. People who did not work on a rice farm had a lower prevalence of infection than those working full time on a rice farm (Table 4). The OR for irrigated villages (reference group: rain-fed villages) was 1.41 (95% BCI: 0.50–3.21).

## Discussion

Our study was specifically designed to capture variations in intensity of infection from village to village and thus eventually identify village-level factors that could be used to improve control strategies. This variation indicates that infection tends to cluster by village, which has been observed with several other parasitic agents [15,21,22]. We found that the prevalence of at least moderate infection could be as high as 23.0% and that of light infection as high as 45.2% in some villages. This demonstrates that despite several years of passive surveillance and mass treatment with praziquantel, *S. japonicum *is still endemic in areas of the Philippines such as Samar Province. It also demonstrates that this variation is most likely due to village-level variables that can not be explained by a crude classification of our villages into the irrigated and non-irrigated categories. We are planning to capture this spatial heterogeneity by updating our initial transmission dynamics model [18] with the data reported here combined with 1-year post-treatment follow-up of study participants. We are also looking at the effect of environmental factors on the presence of *O. hupensis quadrasi *snail colonies in the 50 villages.

Our estimates of prevalences of intensity of infection and their variability in farming households can most likely be generalized across the Samar Province. Only 15.2% of households that had to be replaced were so because their head refused to participate, minimizing the possibility for selection biases. The percentage for the household members who had to be replaced because of refusal to participate was fairly low at 21.1% and is unlikely to introduce selection bias since most of these individuals were children whose mothers refused their participation. Therefore, it is unlikely that the reason for refusal was linked to the risk of *S. japonicum *infection. In addition, 81.3% of all participants provided at least one stool sample. Participants who provided at least one stool sample and those who did not provide any stool sample were similar in respect to age, sex and occupation. The only difference was that a larger proportion of children aged 10 years or less provided at least one stool sample. In the study area, children start school when they are 7 years old and very rarely work outside their homes. Therefore, these children were more likely to be available at the time of the collection of the stool sample. There is no reason to think that individuals who were infected were more or less likely to provide at least one stool sample.

Only about one-third of the participants provided stool samples on three different days. This was a potential complication for data analysis as the sensitivity and specificity of the diagnostic test vary according to the number of samples provided [7,8,23-25]. However, our statistical model allowed us to adjust for this measurement error by estimating the probability of each participant's infection status falling in each of the three epg categories. We also adjusted for the clustering of infection at the village level. To our knowledge, none of these approaches was used in previous studies that described intensity of *S. japonicum *infection in humans.

In our results, we observed higher magnitude of infection among males who are in the 17–40 year age group. In the study region, males who are in that age group are mostly involved in farming activities. This agrees with a prospective study done in a lake region in China which has shown that most water contact occurs in males 18–49 years of age and that human water exposure is occupationally driven [26]. Several other papers reported high intensity of infection among farmers [3,16]. Rice farming requires substantial amounts of standing water for cultivation. In our study, working full time or part time on a rice farm was associated with higher prevalences of infection (and infection intensities) than other occupational groups. The prevalence of *S. japonicum *infection has been reported to be highest among adolescent population [4,11,13,27,28]. This is most likely due to high transmission potential, slow acquisition of resistance, and rapid physical growth in this age group. We found an interaction between sex and age as drivers of the intensity of infection such that females tended to reach a peak of infection earlier (11–16 years old) than males (17–39 years old). This phenomenon has been reported in other studies of *S. japonicum *and may be related to biologically mediated events of puberty and changes in behaviors and social roles at those ages [27,29,30]. Lack of adequate nutrition and unequal distribution of resources to growing females in developing countries may also be factors [13]. We did not find any association between the irrigation status of the villages and *S. japonicum *infection in humans. A better description of the irrigation level of each village with a geographic information system approach is needed to accurately estimate the level of man-made water management on the intensity of infection and also on the presence of colonies of the intermediate host *O. hupensis qusdrasi*.

## Conclusion

Our study is the most comprehensive population-based cross-sectional epidemiological study on *S. japonicum *ever conducted in the Philippines. The results reported here from 50 villages concentrate on the level of human infection and clearly show that a passive treatment approach has failed to control infection in the region. Our results also show that the level of infection is extremely heterogeneous in space and that more geospatial analyses are needed to explain this variation.

## List of abbreviations

BCI: Bayesian Credible Interval

epg: eggs per gram

OR: Odds Ratio

SD: Standard Deviation

## Declaration of competing interests

The author(s) declare that they have no competing interests.

## Authors' contributions

MT contributed to the descriptive data analysis and literature review and wrote the manuscript STMG and HC designed and interpreted the results of the study. HC supervised the data analysis. EB, VT, ROG, PA and RO were responsible for the field data collection for humans and animals. PB and LJ were responsible for conceptualizing and running the data analysis. SR contributed to the interpretation of the results regarding their impact on transmission modeling issues. All authors contributed to interpretation of data and the final version of the manuscript.

## Pre-publication history

The pre-publication history for this paper can be accessed here:



## Figures and Tables

**Table 1:** Description of the 5624 study participants who provided at least one stool sample in 25 rain-fed and 25 irrigated villages of Samar Province, the Philippines, 2003–2004.

Variable		Rain-fed villages (%)	Irrigated villages (%)
Sample size		3,277	2,347
Sex	Males	1,740 (46.9)	1,226 (47.8)
	Females	1,537 (53.1)	1,121 (52.2)
Age distribution	≤ 10 years	638 (19.5)	398 (17.0)
	>10–16 years	973 (29.7)	699 (29.8)
	>16–40 years	944 (28.8)	721 (30.7)
	>40 years	722 (22.0)	529 (22.5)
Occupation*	Works most of the time on a rice farm	1402 (48.9)	1069 (49.7)
	Works sometimes on a rice farm	48 (4.3)	28 (5.2)
	Works on a farm but never on a rice farm	121 (4.6)	85 (4.4)
	Does not work on a farm	482 (18.3)	329 (16.9)
	Does not work on a rice farm, may work on another type of farm	586 (22.2)	438 (22.5)
Number of stool samples provided	1 stool	885 (27.0)	697 (29.7)
	2 stools	1123 (34.3)	770 (32.8)
	3 stools	1269 (38.7)	880 (37.5)

Occupation*: No child less than 10 years old (n = 1036) worked and are therefore excluded from this classification, but it includes children aged more than 10 years old still attending school.

**Table 2:** Village-by-village participation proportion and prevalence of infection in 50 villages of Samar Province, the Philippines, 2003–2004.

Village	No. of Participants	No. of individuals with at least one stool sample (% of total no. of participants)	Proportion with light infection (1–100 epg) with 95% Bayesian credible interval	Proportion with moderate to heavy infection (>100 epg) with 95% Bayesian credible interval
24	114	98 (86.0%)	0.0% (0. 0%–3.1%)	0.0% (0.0%–0.0%)
26	164	141 (86.0%)	0.7% (0. 0%–2.8%)	0.0% (0.0%–0.0%)
2	162	113 (69.8%)	0.9% (0. 0%–4.4%)	0.0% (0.0%–0.0%)
15	152	103 (67.8%)	1.9% (0. 0%–7.8%)	0.0% (0.0%–1.0%)
25	168	149 (88.7%)	2.0% (0.7%–4.7%)	0.0% (0.0%–0.7%)
18	173	121 (69.9%)	2.5% (0.8%–6.6%)	0.0% (0.0%–0.8%)
11	154	119 (77.3%)	3.4% (0.8%–8.4%)	0.8% (0.0%–1.7%)
49	164	142 (86.6%)	3.5% (0.7%–7.8%)	0.0% (0.0%–0.7%)
13	161	125 (77.6%)	4.0% (0.8%–9.6%)	1.6% (0.8%–2.4%)
39	123	88 (71.5%)	4.6% (1.1%–9.1%)	0.0% (0.0%–1.1%)
22	177	151 (85.3%)	6.6% (2.7%–10.6%)	0.0% (0.0%–1.3%)
29	168	143 (85.1%)	7.0% (3.5%–12.6%)	1.4% (0.0%–2.8%)
5	176	145 (82.4%)	7.6% (4.1%–13.1%)	2.1% (0.7%–3.5%)
17	165	138 (83.6%)	8.0% (4.4%–12.3%)	0.0% (0.0%–0.7%)
16	159	148 (93.1%)	8.1% (4.1%–13.5%)	0.0% (0.0%–1.4%)
20	181	138 (76.2%)	8.7% (4.4%–14.5%)	0.0% (0.0%–1.5%)
38	181	168 (92.8%)	9.6% (4.8%–15.6%)	0.0% (0.0%–1.2%)
1	168	125 (74.4%)	12.8% (8.8%–18.4%)	0.0% (0.0%–1.6%)
12	135	113 (83.7%)	14.2% (7.1%–23.0%)	1.8% (0.9%–3.5%)
33	52	48 (92.3%)	14.6% (6.3%–25.0%)	6.3% (2.1%–10.4%)
28	164	142 (86.6%)	15.5% (10.6%–20.4%)	0.7% (0.0%–2.8%)
6	82	75 (91.5%)	16.0% (8.0%–24.0%)	1.3% (0.0%–2.7%)
14	175	143 (81.7%)	16.1% (10.5%–23.1%)	1.4% (0.0%–2.8%)
27	58	45 (77.6%)	17.8% (6.7%–31.1%)	6.7% (0.0%–11.1%)
19	166	118 (71.1%)	17.8% (11.0%–25.4%)	0.9% (0.0%–3.4%)
45	174	135 (77.6%)	18.5% (13.3%–25.2%)	3.0% (0.7%–5.9%)
3	166	155 (93.4%)	18.7% (13.6%– 5.2%)	3.9% (2.6%–6.5%)
35	162	128 (79.0%)	19.5% (14.1%–27.3%)	3.9% (1.6%–6.3%)
7	101	73 (72.3%)	20.6% (13.7%–30.1%)	1.4% (1.4%–5.5%)
8	92	67 (72.8%)	22.4% (13.4%–32.8%)	1.5% (0.0%–4.5%)
21	179	133 (74.3%)	22.6% (15.8%–30.1%)	5.3% (2.3%–9.0%)
32	150	135 (90.0%)	23.0% (16.3%–30.4%)	3.7% (2.2%–5.9%)
41	166	143 (86.1%)	23.1% (16.8%–30.8%)	3.5% (1.4%–6.3%)
40	91	77 (84.6%)	24.7% (16.9%–33.8%)	2.6% (0.0%–7.8%)
4	168	128 (76.2%)	25.0% (18.8%–33.6%)	2.3% (0.8%–6.3%)
34	168	138 (82.1%)	26.8% (19.6%–34.8%)	5.1% (3.6%–8.0%)
9	121	98 (81.0%)	27.6% (20.4%–36.7%)	7.1% (4.1%–12.2%)
46	54	50 (92.6%)	28.0% (20.0%–38.0%)	6.0% (2.0%–10.0%)
50	178	147 (82.6%)	29.3% (22.5%–37.4%)	8.2% (4.8%–11.6%)
42	123	93 (75.6%)	31.2% (23.7%–39.8%)	4.3% (1.1%–7.5%)
23	164	104 (63.4%)	34.6% (26.0%–44.2%)	2.9% (1.0%–7.7%)
30	73	61 (83.6%)	36.1% (26.2%–47.5%)	6.6% (1.6%–13.1%)
31	41	38 (92.7%)	36.8% (26.3%–52.6%)	18.4% (10.5%–26.3%)
47	155	131 (84.5%)	37.4% (29.8%–45.8%)	13.0% (8.4%–18.3%)
37	171	151 (88.3%)	38.4% (31.8%–45.7%)	6.6% (4.0%–12.6%)
44	176	146 (83.0%)	39.0% (31.5%–46.6%)	8.9% (4.8%–13.7%)
36	56	45 (80.4%)	40.0% (28.9%–55.6%)	13.3% (6.7%–22.2%)
48	36	33 (91.7%)	42.4% (30.3%–57.6%)	6.1% (3.0%–15.2%)
10	82	61 (74.4%)	42.6% (31.2%–54.1%)	23.0% (16.4%–31.2%)
43	128	115 (89.8%)	45.2% (36.5%–53.9%)	6.1% (1.7%–13.0%)

**Table 3:** Estimated posterior values of the misclassification between infection categories in humans.

Estimated infection status	Observed classification	Posterior probability of being in the observed infection class given the estimated infection status in % (95% Bayesian credible interval)
Not infected	Not infected	99.3 (98.8 – 99.8)
Not infected	Lightly infected	0.6 (0.1 – 1.1)
Not infected	At least moderately infected	0.1 (0.0 – 0.2)
Lightly infected	Not infected	57.9 (52.2 – 64.1)
Lightly infected	Lightly infected	36.6 (31.8 – 41.1)
Lightly infected	At least moderately infected	5.5 (3.1 – 7.9)
At least moderately infected	Not infected	12.3 (5.1 – 20.7)
At least moderately infected	Lightly infected	26.5 (17.5 – 35.9)
At least moderately infected	At least moderately infected	61.2 (48.3 – 73.6)

**Table 4:** Estimated odds ratios (ORs) of factors associated with the prevalence oflight and at least moderate intensity of infection obtained from hierarchical cumulative logit model adjusted for measurement errorfrom data collected on5624 people living in 50 villages of Samar Province, the Philippines, 2003–2004.

**Variable**		OR* (95% Bayesian credible interval)
**Sex**	Age group	
Male	≤ 10 years	Reference
	>10–16 years	6.10 (3.86 – 9.28)
	>16–40 years	8.76 (6.03 – 12.47)
	>40 years	3.53 (2.37 – 5.15)
Female	≤ 10 years	Reference
	>10–16 years	8.59 (4.74 – 14.28)
	>16–40 years	5.98 (3.53 – 9.61)
	>40 years	3.46 (2.02 – 5.55)
**Age group**	Sex	
≤ 10 years	Female	Reference
	Male	2.31 (1.40 – 3.61)
>10–16 years	Female	Reference
	Male	1.66 (1.01 – 2.61)
>16–40 years	Female	Reference
	Male	3.39 (2.32 – 4.80)
>40 years	Female	Reference
	Male	2.37 (1.54 – 3.58)
**Occupation**		
Works most of the time on a rice farm		Reference
Works sometimes on a rice farm		1.32 (0.82 – 2.02)
Works on a farm but never on a rice farm		0.42 (0.15 – 0.87)
Does not work on a farm		0.55 (0.30 – 0.90)
Does not work on a rice farm, may work on another type of farm		0.52 (0.37 – 0.72)
**Irrigation**		
Rain-fed		Reference
Irrigated		1.41 (0.50 – 3.21)

OR*: the OR compares individuals at least lightly infected (>0 epg) to individuals not infected (0 epg) and individuals moderately or heavily infected (>100 epg) to individuals lightly infected or not infected (0–100 epg).
